# Factors related to cancer screening behaviors

**DOI:** 10.4178/epih.e2018011

**Published:** 2018-03-29

**Authors:** Boyoung Choi, Tae Rim Um, Kwang-Soo Lee

**Affiliations:** 1 Department of Public Health and Medical Administration, Dongyang University, Yeongju, Korea; 2 Department of Health Administration, Yonsei University Graduate School, Wonju, Korea; 3 Department of Health Administration, Yonsei University College of Health Sciences, Wonju, Korea

**Keywords:** Early detection of cancer, Demography, Propensity score, Korean Community Health Survey

## Abstract

**OBJECTIVES:**

This study aimed to investigate the factors related to cancer screening behaviors (CSB).

**METHODS:**

The 2014 Korean Community Health Survey used for analysis. The dependent variable was CSB, and the independent variables were demographic, health behavioral, and regional factor. Propensity score matching (PSM) used to control health behavior and regional factors, which were influencing CSB. For statistical analysis, chi-square test and logistic regression analysis used.

**RESULTS:**

Logistic regression analysis after PSM showed that gender, age, marital status, educational level, monthly household income, employment type, alcohol drinking, smoking, body mass index group, chronic disease, and subjective health status influenced the CSB, there were statistical differences.

**CONCLUSIONS:**

To improve cancer screening (CS), it is necessary to educate individuals on the need for CS and to carry out a personalized CS program based on an individual’s demographic status and health behavior.

## INTRODUCTION

Cancer is a disease responsible for millions of deaths worldwide. According to the World Health Organization (WHO) [1], 14 million people died of cancer in 2012. In Korea, the incidence has reached 250 per 100,000 population since 2008 [2], with 79,000 cancer-related deaths reported in 2016 [3]. As of 2016, the number of cancer-related deaths per 100,000 population by cancer types, in descending order, was lung cancer (n=52.2), liver cancer (n=31.5), and gastric cancer (n= 20.8) among men and lung cancer (n= 18.1), colon cancer (n= 14.6), and liver cancer (n= 11.6) among women [4].

The onset of cancer causes physical and mental suffering in the patient and their family, while increasing economic burden. From a national perspective, it causes economic losses due to loss of human resources and decreased productivity, increasing national healthcare expenditure [5].

Cancer treatment outcome varies significantly by cancer stage. The 5-year relative survival rate (RSR) for major cancers (gastric, colorectal, breast, cervical, prostate, and thyroid cancers), excluding lung cancer, is approximately 90.0-100.0% when the cancer is localized to a single organ. However, in cases of distant metastasis, the 5-year RSR for major cancers, excluding thyroid cancer, is only 5.5-36.4% [6]. As shown, the treatment effect of cancer can link directly to early detection of cancer.

The WHO reported that prevention of cancer is possible through changes in dietary habits and lifestyle, while the distress by cancer can reduce through early cancer detection, accurate diagnosis and effective treatment [7]. Primary prevention through changes in lifestyle are difficult since it relies on each individual. Therefore, it is important to utilize cancer screening (CS), a secondary prevention, to seek early cancer detection. For this, most countries have implemented national CS programs, while also using various promotional efforts for early cancer detection. In Korea, two type of national CS programs have for low-income families and the five major cancers for civilian. Additionally, CS is also offered by private insurance companies, meaning CS is available in various ways [8].

However, despite various nationwide efforts in promoting such programs, the rate of participation in the national CS for gastric, liver, colorectal, breast, and cervical cancer in Korea was only 45.3, 43.9, 32.9, 49.0, and 40.5%, respectively, in 2011 [9]. Various studies have followed to identify the reasons for such low CS participation rate. CS-related studies can divide into two major categories: (1) studies on the individual respondents that examined the cancer screening behaviors (CSB) [8,10] and specific CS rates [11-13] and (2) studies on environmental factors that examined the health screening behaviors of individuals in the urban versus rural settings [14]. However, most studies examined a specific cancer or region, which makes it difficult to identify the factors associated with overall CS for the entire population of Korea. To increase the rate of participation in CS, it is important to identify not only the environmental and health behavior factors that influence CS, but also the CS-related demographic characteristics of each individual to provide cancer education and screening services that are customized by various characteristics and social classes.

Accordingly, this study aimed to identify the demographic characteristics that show differences in CSB in presenting the basic data that can help increase the rate of participation in CS. For the more objective analysis of CSB, this study used propensity score matching (PSM) of the confounding factors such as health behavioral and regional factors.

## MATERIALS AND METHODS

### Research model

**Figure 1.**
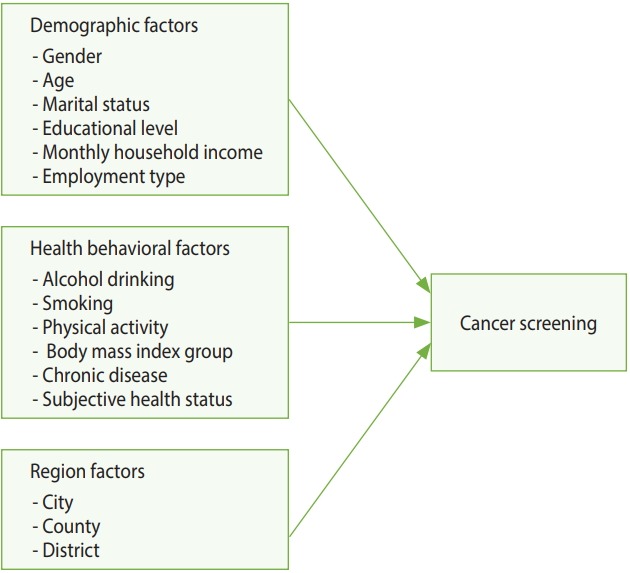
The conceptual model of this study.

Figure 1 shows the model used in this study. Analysis performed with the demographic, health behavior, and regional characteristics as independent variables and CSB as the dependent variable.

### Materials and respondents

This study based on the data from the 2014 Korean Community Health Survey (KCHS). KCHS is a Korea Centers of Disease Control and Prevention (KCDC)-sponsored survey. This survey of adults aged ≥ 19 years conducted annually between August and October by 253 public health centers throughout Korea. Among 228,712 adults who were included in this survey, 136,627 selected for the present study after PSM and excluding 19-29 age group who were not included in the national CS program and those with missing responses or values.

### Variables

#### Dependent variable

CSB was set as the dependent variable, which was divided into two categories: “yes,” if the individual participated in a CS (national or private CS) in the past 2 years, and “no”.

#### Independent variables

Demographic, health behavior, and regional characteristics were set as independent variables. The demographic characteristics include gender, age, marital status, education level, household income, and employment status [8,11,12,14-16]. The health behavior factors included drinking, smoking, physical activity, obesity, chronic disease, and subjective health status [8,11,15,17]. Regional characteristics were divided into city, county, and district. Sejong city excluded from the analysis as it might influence by confounding factors and biases [18,19].

### Analysis method

PSM applied to control the health behavior and regional characteristics, which may be present besides the variables of interest (demographic characteristics). Chi-square analysis performed to identify the differences between before and after PSM CSB by each factor. Additionally, logistic regression analysis performed to identify the influencing factors of CSB and their degree of influence.

#### Propensity score matching

This study used PSM to control any confounding factors. Generally, studies that compare two populations use non-randomized sampling design, which present problems with convenience sampling and the results being overestimated or underestimated. Accordingly, the PSM method introduced, where the variables that can act as confounding factors are preselected in the design stage and calculated as covariates to closely match the treated and control groups [20-22].

PSM analysis involved the following processes: (1) the propensity scores (PS) of the treated and control groups were estimated. The dependent variable in the present study was CSB and potential influencing health behavior factors and regional characteristics introduced as covariates to estimate the PS; (2) estimated PS were compared to create matching treated and control groups with similar PS. The study used the Greedy matching method, which uses a caliper to set a specific range of PS from the center of the treated group and select individuals in the control group whose scores are closest to that range [20]. Although there is no set tolerance for the caliper, 0.01-0.00001 is typically used. We used 1:1 nearest neighbor matching with a caliper of 0.0001. As a result, 136,672 individuals were matched; (3) after performing PSM, the treated and control groups were assessed to ensure that they were properly matched. In this study, matching results were verified using standardized differences and graphs of the covariates in the treated and control groups. Subsequently, chi-square analysis performed on the matched individuals [23].

#### Logistic regression analysis

To identify the influence of demographic factors on CBS before and after PSM, a logistic regression analysis performed. PSM performed using SPSS version 23.0 (IBM Corp., Armonk, NY, USA), while logistic regression analysis was performed using SAS version 9.4 (SAS Institute Inc., Cary, NC, USA).

## RESULTS

### Descriptive analysis of the general characteristics of the respondents

Generally, differences noted before and after matching are analyzed to determine whether PSM produced well-matched results. Table 1 shows the results of the analysis on the differences between the characteristics of respondents before and after matching. Before matching, there were 115,665 individuals from the group who participated in CS and 73,010 from the group who did not participate. After introducing health behavior factors and regional characteristics as covariates and performing 1:1 matching by PS, results revealed a total of 68,336 matching individuals from the treated and control group. Variables that showed significant differences before matching (drinking, smoking, physical activity, obesity, chronic disease, subjective health status, and regional characteristics) did not show statistically significant differences after matching, indicating that PSM produced well-matched results.

**Figure 2.**
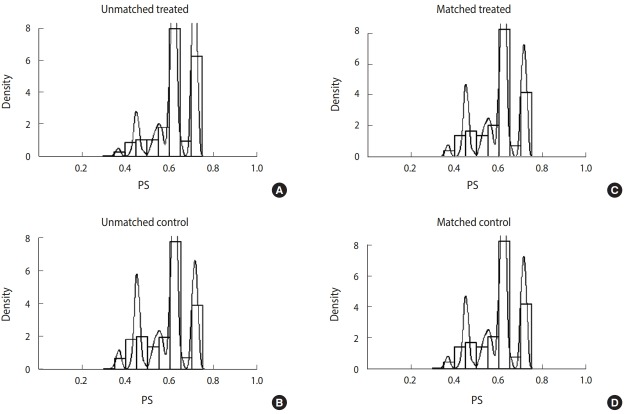
Comparison of propensity score (PS) distribution before and after propensity score matching, treated: cancer screening “yes”, control: cancer screening “no”. (A) Unmatched treated. (B) Unmatched control. (C) Matched treated. (D) Matched control.

Figure 2 shows the distribution of unmatched and matched PS of the treated and control groups as histograms for determining whether PSM produced well-matched results. The distribution of PS of the groups who did and did not participate in CS became similar after matching.

### Logistic regression analysis with cancer screening status as the dependent variable

Table 2 shows the results of logistic regression analysis with CBS status before and after PSM as the dependent variable and demographic, health behavior, and regional variables as independent variables (Wald test for global null hypothesis: χ^2^ = 6,722.866, p< 0.001 [before PSM]; χ^2^ = 9,516.227, p< 0.001 [after PSM]). The final model was selected based on Akaike information criterion and c-statistics, but the model was determined to be unfit in the Hosmer-Lemeshow test (p< 0.001). It is believed that HosmerLemeshow test may appear to be significant because the differences between the predicted and observed values are small with extremely large sample size [24].

In the analysis before PSM, men were less likely (39.0%) to participate in CS, while those aged 50-69 years were more likely and 30-49 were less likely to participate in CS than those aged ≥ 70 years. Compared to unmarried respondents, married respondents were 2.56 times, while separated, divorced, or widowed respondents were 1.62 times more likely to participate in screening. Moreover, those with lower education level and monthly income were less likely to participate in CS. Compared to being inoccupation, employers or self-employed person were 1.16 times more likely and salaried workers were 1.35 times more likely to participate in CS.

Moreover, the odds ratio (OR) of participating in CS was 1.15 and 1.33 times higher in current drinkers and people exercising, respectively, while those categorized as current smokers were 35.0% less likely to participate in CS. Relative to the normal body mass index (BMI) group, those in the underweight and obese groups were less likely to participate in CS (9.0 and 4.0%, respectively). Those who has chronic disease were 1.26 times more likely to participate in CS. Respondents whose health status was “average” were more likely to participate in CS than those whose response was “healthy.” Those who reside in a district were more likely to participate in CS than those who reside in a city or country.

In the analysis after PSM, men were less likely to participate in CS (38.0%), while those aged 50-69 years were more likely and 30-49 were less likely to participate in CS than those aged ≥ 70 years. Compared to unmarried respondents, married respondents were 2.50 times and separated, divorced, or widowed respondents were 1.62 times more likely to participate in screening. Moreover, those with lower education level and monthly income were less likely to participate in CS. Employers or self-employed person were 1.15 times and salaried workers were 1.36 times more likely to participate in CS than unemployed people were.

Moreover, the OR of participating in CS was 1.07 and 1.30 times higher in current drinkers and current smokers, respectively, while people exercising were 6.0% less likely to participate in CS. Relative to the normal BMI group, those in the underweight and obese groups were slightly less likely to participate in CS. Those who have chronic disease were 20.0% less likely to participate. Respondents whose subjective health status was “unhealthy” were 1.11 times more likely to participate in CS than those whose response was “average.”

## DISCUSSION

### Data and method

This study used the data from the 2014 KCHS. Previous CS studies focused mostly on a specific cancer [11,13,14] or some residents in a specific region [8,10,14,15,25], making it difficult to identify the factors associated with overall CS for the entire population of Korea. Accordingly, the present study was significant as it used the data from a standardized survey system that allowed comparisons between regions to examine the overall nationwide CS status.

PSM uses PS to balance the covariates observed in the respondents from each group used in the study, creating a setting similar to a randomized study [26]. This is a method that had not been widely used in previous studies on the influencing factors of CSB; thus, the present study has the advantage of minimizing selection bias and confounding factors by using PSM for a more definitive identification of the influencing factors of CSB.

### Study results

A logistic regression analysis performed before and after PSM. Smoking, chronic disease, and region showed differences before and after PSM.

The logistic regression analysis results before and after PSM were similar among demographic characteristics. In the present study, women showed higher CS rates than men, which was consistent with the results of previous studies [5,8,25,27-29], but inconsistent with other studies on the influencing factors of gastric CS, which reported slightly higher screening rates among men [30,31]. This is explained by lack of time due to greater social participation by men, their lack of awareness about CS, and decrease willingness to participate in the screening. As programs included breast and cervical CS that apply only to women, women may have more opportunities to participate in the screening [25,27] Hence, CS rates among men may possibly increase if CS items include other types of cancers that only occur in men, such as prostate cancer, and actively utilize workplace screening.

Aged people were more likely to participate in CS, which was consistent with the results of previous studies [5,11,25,27,29,32]. The older people are more susceptible to chronic diseases, which increases their opportunity for healthcare utilization and in turn would increase their awareness and participation in CS as they acquire information about health management and screening [27]. Moreover, the OR of CS was relatively low among individuals aged ≥ 70 years probably due to the low demand for and benefits from CS, which would lower the likelihood of their participation in CS [25].

With regard to marital status, most studies [8,11,15,25,27,30, 32-34] showed that the CS rate was higher among married individuals than those who were unmarried, a tendency that was consistent with the findings in the present study. In this study, respondents who were married as well as widowed or divorced showed higher CS rates than those who were unmarried. This may be due to the support and interest from the spouse or family members aside from being an influencing factor of CS. Respondents with lower education level and monthly household income were less likely to participate in CS, which was consistent with the results of previous studies [8,11,15,29,30,32,33]. Therefore, these results suggest the need for policy-based efforts to promote equality in CS.

In most studies, employment status did not show significant differences or was not included in the study at all [5,8,10,11,25,29,34]. In the present study, respondents who were employed (salaried workers, self-employed, and unpaid homemakers) were more likely to participate in CS than those who were unemployed, which was contrary to the study on cervical and breast cancers by Kim et al. [35]. The study by Kim et al. [35] was conducted only in women, whose greater time flexibility may have a greatly influenced them to participate in CS. Moreover, when targeting both men and women, improving healthcare accessibility by providing workplace screening and increasing the opportunity to acquire information about CS may influence the likelihood of participating in CS.

The result of current drinking was similar and current smokers were 35.0% less likely, but 1.30 times more likely to participate in CS before and after PSM. In previous studies, health behavior factors, such as drinking and smoking, showed conflicting influence on CS [5,28,30,33,34]. Drinking and smoking are well-known risk factors of cancer. Because current drinkers and smokers already recognized them as undesirable health behaviors, they may have been more likely to participate in CS. These factors were successfully adjusted by PSM. Before PSM, the underweight and obese groups were less likely to participate in CS than the normal group, whereas after PSM, the same groups were more likely to participate in CS. These results were consistent with the results of Park et al. [34] and Fagan et al. [36] studies on men with colorectal and prostate cancer, but inconsistent with the results of Fagan et al. [36] study on cervical cancer. The subjects who were underweight or obese may have viewed themselves as being unhealthy, which may have increased their participation in CS.

Chronic disease and subjective health status also showed differences before and after PSM. The results after PSM were consistent with those of existing studies, which reported that patients with chronic diseases have higher healthcare utilization due to regular hospital visits, which increases their participation in CS [8,34,36]. This demonstrated that this factor was well adjusted. With regard to subjective health status, previous studies on CS-related factors [8], cancer survivors [27,33], and CS in South Gyeongsang Province [28] did not show significant differences. In the study, respondents whose subjective health status after PSM was categorized as “unhealthy” were 1.11 times more likely to participate in CS. It is believed that they may have participated in CS at a higher rate due to concerns about their own health.

Respondents who resided in cities or counties were more likely to participate in CS than those residing in districts before PSM, but these respondents did not show significant differences after PSM. Lack of regional disparity may be explained by the fact that because of the availability of CS at various medical institutions, the problem of healthcare accessibility may have been partially resolved [25].

The policy implications based on the findings of this study are as follows: (1) to increase the rate of participation in CS, the intention to participate in CS must be increased. CS intention can be viewed as a strong influencing factor of CSB [8]. To increase CS intention in individuals, it is important to provide education to inform the individuals on the importance of early cancer detection through screening to prevent cancer and improve the survival rate after cancer. Therefore, the rate of participation in CS should be increased by improving the personal CS intention and motivation through systematic educational programs and promotions; (2) the rate of participation in CS showed differences based on demographic factors, such as education level, monthly household income, and employment status. To resolve this disparity, it is necessary to expand the scope of free screening for most common cancers and create an environment that would make it easier for people to participate in CS, such as providing paid vacation time. Additionally, it is also necessary to implement CS-related promotion and education for the population with low income and low education level; and (3) since cancer is a disease that requires regular screening, it is important to increase the rate of re-screening. According to a study by Kim [37], higher patient satisfaction in CS resulted in higher cancer re-screening intention. For this, the quality of CS must be improved and the existing policies and systems must be upgraded to make them more diverse and customized based on the demographic characteristics of individuals.

The following are the limitations of this study: (1) CS status in KCHS data included national and private CS programs. As a result, the scope and characteristics of CS may vary between individuals; (2) the study used a cross-sectional survey, causality cannot be determined. In the future, it would be necessary to use cohort or panel data to investigate the causal relationships between the variables; (3) other known influencing factors of CS that were not provided in the original data could not be examined, which include family [30,38] and personal history [29,30] of cancer. Future studies should conduct analysis with inclusion of all known influencing factors of CS, so that these factors can be identified more clearly; (4) KCHS collected data by 1:1 interviews through home visits. Therefore, biases related demographic variables and recall bias might be introduced. Therefore, this issue should be addressed by conducting analysis that is linked to CS-related healthcare utilization and access to medical records.

The results of this study showed that gender, age, marital status, education level, household income, drinking, smoking, obesity, chronic disease, and subjective health status were the variables that influenced CSB.

CS is important for early detection of cancer to increase survival rate. Therefore, it is necessary to raise awareness about the importance of CSB through education and increase the rate of participation in CS through various promotional channels. Moreover, since cancer is a disease that requires regular screening, it is important to increase the re-screening rate, which may require development and expansion of individualized CS programs that are linked to the demographic and health behavior factors of individuals, instead of providing generic CS program.

This study is significant as it used the data that were designed to allow regional comparisons in identifying the factors influencing CSB in the entire population of Korea. Moreover, the study performed more definitive analysis by controlling any confounding factors using PSM.

## Figures and Tables

**Table 1.** General characteristics of study variables by cancer screening

Variables	Cancer screening					
	Before PSM			After PSM		
	Yes (n=115,665)	No (n=73,010)	χ^2^	Yes (n=68,336)	No (n=68,336)	χ^2^
PS matching variables						
Alcohol drinking						
Current drinker	77,853 (67.3)	50,813 (69.6)		46,820 (68.5)	46,820 (68.5)	0.000
Non-drinker	37,812 (32.7)	22,197 (30.4)	108.053^***^	21,516 (31.5)	21,516 (31.5)	
Smoking						
Current smoker	97,025 (83.9)	52,270 (71.6)	4,094.415^***^	51,946 (76.0)	51,946 (76.0)	0.000
Non-smoker	18,640 (16.1)	20,740 (28.4)		16,390 (24.0)	16,390 (24.0)	
Physical activity						
Yes	100,595 (87.0)	60,505 (82.9)	602.557^***^	57,181 (83.7)	57,181 (83.7)	0.000
No	15,070 (13.0)	12,505 (17.1)		11,155 (16.3)	11,155 (16.3)	
Body mass index						
Underweight	4,655 (4.0)	3,779 (5.2)	206.896^***^	3,261 (4.8)	3,404 (5.0)	3.636
Normal	50,812 (43.9)	32,021 (43.9)		30,089 (44.0)	30,089 (44.0)	
Overweight	30,270 (26.2)	17,727 (24.3)		16,908 (24.7)	16,908 (24.7)	
Obesity	29,928 (25.9)	19,483 (26.7)		18,078 (26.5)	17,935 (26.2)	
Chronic disease						
Yes	61,227(52.9)	46,956 (64.3)	2,369.459^***^	42,537 (62.2)	42,537 (62.2)	0.000
No	54,438(47.1)	26,054 (35.7)		25,799 (37.8)	25,799 (37.8)	
Subjective health status						
Healthy	38,027 (32.9)	26,767 (36.7)	300.865^***^	24,472 (35.8)	24,615 (36.0)	1.124
Average	51,674 (44.7)	31,300 (42.9)		29,329 (42.9)	29,329 (42.9)	
Unhealthy	25,964 (22.4)	14,943 (20.5)		14,535 (21.3)	14,392 (21.1)	
Region						
City	33,524 (29.0)	21,372 (29.3)	102.252^***^	19,924 (29.2)	19,924 (29.2)	0.000
County	39,911 (34.5)	23,638 (32.4)		22,409 (32.8)	22,409 (32.8)	
District	42,230 (36.5)	28,000 (38.4)		26,003 (38.1)	26,003 (38.1)	
PS non-matching variables						
Gender						
Men	48,819 (42.2)	39,166 (53.6)	2,352.684^***^	31,576 (46.2)	34,969 (51.2)	337.168^***^
Women	66,846 (57.8)	33,844 (46.4)		36,760 (53.8)	33,367 (48.8)	
Age (yr)						
30-39	12,720 (11.0)	20,789 (28.5)	11,063.150^***^	8,572 (12.5)	18,904 (27.7)	6,138.021^***^
40-49	26,747 (23.1)	16,162 (22.1)		17,514 (25.6)	14,832 (21.7)	
50-59	30,763 (26.6)	13,297 (18.2)		18,031 (26.4)	12,512 (18.3)	
60-69	24,537 (21.2)	9,006 (12.3)		13,017 (19.0)	8,732 (12.8)	
≥70	20,898 (18.1)	13,756 (18.8)		11,202 (16.4)	13,356 (19.5)	
Marital status						
Married	94,512 (81.7)	51,344 (70.3)	6,955.404^***^	55,990 (81.9)	48,321 (70.7)	3,782.788^***^
Separated/divorced/widowed	17,710 (15.3)	12,527 (17.2)		9,905 (14.5)	11,922 (17.4)	
Unmarried	3,443 (3.0)	9,139 (12.5)		2,441 (3.6)	8,093 (11.8)	
Educational level						
Primary school	30,281 (26.2)	16,621 (22.8)	1,554.073^***^	16,138 (23.6)	16,113 (23.6)	530.219^***^
Middle school	16,724 (14.5)	7,689 (10.5)		9,344 (13.7)	7,297 (10.7)	
High school	36,077 (31.2)	23,811 (32.6)		22,155 (32.4)	21,859 (32.0)	
College	9,525 (8.2)	8,534 (11.7)		6,060 (8.9)	7,817 (11.4)	
University	18,936 (16.4)	14,024 (19.2)		12,105 (17.7)	13,026 (19.1)	
Graduated school	4,122 (3.6)	2,331 (3.2)		2,534 (3.7)	2,224 (3.3)	
Monthly household Income (10^3^ KRW)						
<1,000	24,719 (21.4)	16,129 (22.1)	621.613^***^	13,541 (19.8)	15,426 (22.6)	707.748^***^
1,000-<2,000	20,314 (17.6)	12,760 (17.5)		11,419 (16.7)	11,940 (17.5)	
2,000-<3,000	20,756 (17.9)	15,156 (20.8)		12,515 (18.3)	14,000 (20.5)	
3,000-<4,000	18,178 (15.7)	12,199 (16.7)		11,211 (16.4)	11,315 (16.6)	
4,000-<5,000	12,643 (10.9)	7,277 (10.0)		7,855 (11.5)	6,771 (9.9)	
≥5,000	19,055 (16.5)	9,489 (13.0)		11,795 (17.3)	8,885 (13.0)	
Employment type						
Employer and self-employed	25,594 (22.1)	15,613 (21.4)	126.180^***^	15,469 (22.6)	14,322 (21.0)	102.067^***^
Salary	42,680 (36.9)	28,815 (39.5)		26,860 (39.3)	26,327 (38.5)	
Inoccupation	47,391 (41.0)	28,582 (39.1)		26,007 (38.1)	27,687 (40.5)	

Values are presented as number (%). PSM, propensity score matching; PS, propensity score; KRW, Korean won.

*** p<0.001.

**Table 2.** Results of the logistic regression analysis

Variables	Before PSM^1^	After PSM^2^
Gender (ref: women)		
Men	0.61 (0.59, 0.63)^***^	0.62 (0.60, 0.63)^***^
Age (ref: ≥70, yr)		
30-39	0.31 (0.29, 0.32)^***^	0.30 (0.28, 0.32)^***^
40-49	0.78 (0.75, 0.82)^***^	0.76 (0.72, 0.80)^***^
50-59	1.12 (1.08, 1.17)^***^	1.07 (1.03, 1.12)^**^
60-69	1.50 (1.45, 1.55)^***^	1.45 (1.39, 1.51)^***^
Marital status (ref: unmarried)		
Married	2.56 (2.45, 2.68)^***^	2.50 (2.38, 2.63)^***^
Separated/divorced/widowed	1.62 (1.54, 1.71)^***^	1.62 (1.53, 1.72)^***^
Educational level (ref: graduated school)		
Primary school	0.65 (0.61, 0.69)^***^	0.66 (0.62, 0.71)^***^
Middle school	0.77 (0.73, 0.83)^***^	0.78 (0.73, 0.84)^***^
High school	0.74 (0.70, 0.79)^***^	0.75 (0.70, 0.80)^***^
College	0.82 (0.77, 0.87)^***^	0.81 (0.76, 0.87)^***^
University	0.87 (0.82, 0.93)^***^	0.88 (0.83, 0.94)^***^
Monthly household income (ref: ≥5,000, 10^3^ KRW)		
<1,000	0.70 (0.67, 0.73)^***^	0.69 (0.66, 0.72)^***^
1,000-<2,000	0.73 (0.70, 0.76)^***^	0.71 (0.68, 0.74)^***^
2,000-<3,000	0.75 (0.72, 0.77)^***^	0.74 (0.71, 0.77)^***^
3,000-<4,000	0.82 (0.79, 0.85)^***^	0.81 (0.78, 0.85)^***^
4,000-<5,000	0.93 (0.89, 0.96)^***^	0.92 (0.88, 0.96)^***^
Employment type (ref: inoccupation)		
Employer and self-employed	1.16 (1.13, 1.20)^***^	1.15 (1.12, 1.19)^***^
Salary	1.35 (1.31, 1.39)^***^	1.36 (1.32, 1.40)^***^
Alcohol drinking (ref: non-drinker)		
Current drinker	1.15 (1.13, 1.18)^***^	1.07 (1.05, 1.10)^***^
Smoking (ref: non-smoker)		
Current smoker	0.65 (0.64, 0.67)^***^	1.30 (1.26, 1.34)^***^
Physical activity (ref: no)		
Yes	1.33 (1.29, 1.37)^***^	0.94 (0.91, 0.97)^***^
Body mass index (ref: normal)		
Underweight	0.91 (0.87, 0.95)^***^	1.10 (1.05, 1.16)^***^
Overweight	1.00 (0.97, 1.02)	0.96 (0.93, 0.99)^**^
Obesity	0.96 (0.93, 0.98)^***^	1.05 (1.02, 1.08)^***^
Chronic disease (ref: no)		
Yes	1.26 (1.23, 1.30)^***^	0.80 (0.77, 0.82)^***^
Subjective health status (ref: average)		
Healthy	0.94 (0.92, 0.96)^***^	1.02 (0.99, 1.05)
Unhealthy	1.01 (0.98, 1.04)	1.11 (1.07, 1.14)^***^
Region (ref: district)		
City	1.08 (1.06, 1.11)^***^	1.01 (0.99, 1.04)
County	1.13 (1.10, 1.16)^***^	1.00 (0.98, 1.03)

Values are presented as odds ratio (95% confidence interval). PSM, propensity score matching; KRW, Korean won.

1 c-statistics=0.683, Hosmer-Lemeshow goodness-of-fit test p<0.001.

2 c-statistics=0.653, Hosmer-Lemeshow goodness-of-fit test p<0.001.

** p<0.01,

*** p<0.001.

## SUPPLEMENTARY MATERIALS

Supplementary Material: Korean version is available at http://www.e-epih.org/.
