# Supplementary material for: Factors related to cancer screening behaviors
Source: Epidemiol Health. 2018 Mar 29;40:e2018011. doi: 10.4178/epih.e2018011 (PMC5968203; doi:10.4178/epih.e2018011)
Supplement: Supplementary file 1 [file epih-40-e2018011-supplementary.pdf]

Volume: 40, Article ID: e2018011
https://doi.org/10.4178/epih.e2018011

Factors related to cancer screening behaviors

Boyoung Choi[1], Tae Rim Um[2], Kwang-Soo Lee[3]

[1]Department of Public Health and Medical Administration, Dongyang University, Yeongju, Korea; [2]Department of Health Administration, Yonsei University Graduate School, Wonju, Korea; [3]Department of Health Administration, Yonsei University College of Health Sciences, Wonju, Korea

Abstract

Objectives: This study aimed to investigate the factors related to cancer screening behaviors(CSB).

Methods: The 2014 community health survey was used for analysis. The dependent variable was CSB, and the independent variables were demographic, health behavioral, and regional factor. Propensity score matching (PSM) was used to control health behavior and regional factors, which were influencing CSB. For statistical analysis, chi-square test and logistic regression analysis were used.

Results: Logistic regression analysis after PSM showed that sex, age, marital status, educational level, monthly household income, employment type, alcohol drinking, smoking, body mass index(BMI) group, chronic disease, and subjective health status influenced the CSB, there were statistical differences.

Conclusions: To improve cancer screening, it is necessary to educate individuals on the need for cancer screening and to carry out a personalized cancer screening program based on an individual's demographic status and health behavior.

Keywords: Early Detection of Cancer, Demography, Propensity Score, Korean Community Health Survey

Volume: 40, Article ID: e2018011
https://doi.org/10.4178/epih.e2018011

Volume: 40, Article ID: e2018011
https://doi.org/10.4178/epih.e2018011

INTRODUCTION

암은 세계적으로 수백만 명의 사망자를 발생시키는 질병이다. 세계보건기구(WHO, 2017)[1] 에 의하면 2012년 1,400만 명이 암으로 사망하였으며, 우리나라에서는 2008년 이후 인구 10만 명 당 250여 명의 신규 암 환자가 발생하였고[2], 2016년 7만 9천여 명이 암으로 인해 사망하였다[3]. 우리나라의 2016년 인구 10만 명당 암 종별에 따른 사망자 수는 남자의 경우 폐암(52.2명), 간암(31.5명), 위암(20.8명) 순으로 나타났고, 여자의 경우에는 폐암(18.1명), 대장암(14.6명), 간암(11.6명) 순으로 나타났다[4].

암 발병은 환자와 가족의 신체적·정신적 고통을 초래하고, 경제적 부담을 증가시킨다. 또한 국가적 차원에서는 인적 자원의 유실 및 생산성 감소로 인한 경제 적 손실, 국민의료비 증가 등의 문제를 발생시킨다[5].

암으로 인한 위해를 최소화하기 위해서는 암의 예방과 치료가 중요하다. 암의 치료 성과는 병기에 따라 큰 차이가 있는데 암이 한 장기에만 국한되어 있는 경우 간암과 폐암을 제외한 주요 암종(위암, 대장직장암, 유방암, 자궁경부암, 전립선암, 갑상선암)의 5년 상대생존율은 약 90.0%~100.0%이지만 멀리 떨어진 다른 장기에 암이 전이된 원격 전이 단계에서 갑상선암을 제외한 주요 암종의 5년 상대생존율은 36.4%~5.5%에 불과하였다[6]. 이와 같이 암 치료의 효과는 암의 조기 발견과 직결된다고 할 수 있다.

세계보건기구(WHO)는 식이습관 및 생활방식의 변화를 통해 암을 예방할 수 있고, 암의 조기발견과 정확한 진단 및 효과적인 치료방법을 통해 암으로 인한 고통을 경감시킬 수 있다고 보고하였다[7]. 식습관 및 생활방식을 통한 1차 예방 방법은 개인의 의지에 따라 좌우되기 때문에 관리가 어렵다. 따라서 2차 예방 방법인 암 검진을 통해 암을 조기에 발견하는 것이 중요하다. 이를 위해 대부분의 국가에서는 국가적 차원의 암 검진 프로그램을 마련하여 암 검진 사업을 시행하고 있으며, 암 검진에 대한 다양한 홍보를 통해 암 조기 발견을 위해 노력하고 있다. 우리나라의 경우 저소득층을 대상으로 하는 국가 암조기검진사업과 일반인을 대상으로 위암, 간암, 대장암, 유방암, 자궁경부암의 5대 암에 대한 검진 사업이 있다. 그 외 민간보험회사에서 실시하는 암 검진이 있으며, 다양한 경로를 통하여 암 검진을 받을 수 있다[8].

그러나 국가차원의 다양한 사업 홍보에도 불구하고 특히, 국가 암 검진사업의 전체 암 검진 수검률은 2011년 위암 45.3%, 간암 43.9%, 대장직장암 32.9%, 유방암 49.0%, 자궁경부암 40.5% 등 낮은 수준에 머무르고 있다[9]. 이에 암 검진 수검률이 낮은 원인을 밝히기 위하여 다양한 연구가 진행되었다. 암 검진에 대한 연구는 크게 두 가지로 나누어 볼 수 있다. 첫째는 수진자 개인에 대한 연구로 암 검진 수진행동에 대한 연구[8, 10], 특정 암의 수검률에 대한 연구[11-13]가 있으며, 둘째로는 환경적 요인에 대한 연구로 도시지역과 농촌지역의 건강검진 수진행동에 관련된 연구[14]가 있다. 그러나 대부분의 연구는 특정 암 또는 일부 지역을 대상으로 조사되어 우리나라 국민의 전반적인 암 검진 수진요인을 파악하는데 어려움이 있다. 암 검진 수검률을 높이기 위해서는 검진에 영향을 미치는 환경 및 건강행태 요인뿐만 아니라 수진자 개인의 암 검진 관련 인구사회학적 특성을 파악해 특성별, 계층별로 암 검진 교육과 맞춤형 검진을 제공하는 것이 필요하다.

이에 연구를 통해 암 검진 수진행동에 차이를 보이는 인구사회학적 특성을 파악하여 수검률 향상에 도움이 되는 기초자료를 제시하고자 한다. 본 연구에서는 암 검진 수검행동을 보다 객관적으로 분석하기 위해 건강행태, 지역 특성에 대해 성향점수 매칭(propensity score matching, PSM)을 사용하여 교란요인을 통제하였다. 본 연구의 구체적인 연구 목적은 암 검진 수진행동에 미치는 인구사회학적 특성요인의 영향을 파악하는 것이다.

Volume: 40, Article ID: e2018011
https://doi.org/10.4178/epih.e2018011

MATERIALS AND METHODS

1. 연구 모형

본 연구 모형은 <Figure 1>과 같으며, 인구사회학적 특성, 건강행태, 지역 특성을 독립변수로 하고 암 검진 수진행동을 종속변수로 하여 분석을 실시하였다.

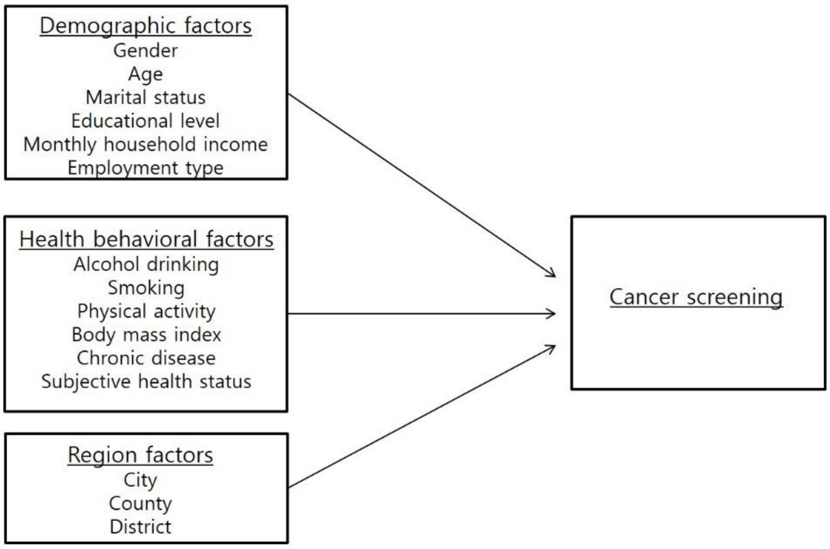

Figure 1. The conceptual model of this study.

2. 연구자료 및 대상

본 연구는 2014년 지역사회건강조사 자료를 바탕으로 이루어졌다. 지역사회건강조사는 질병관리본부 주관 하에 전국 253개 보건소가 매년 8월에서 10월까지 실시하는 것으로 만 19세 이상의 성인을 대상으로 한다. 본 연구에서는 조사에 포함된 총 228,712명 중 국가 암 검진 사업의 검진 대상자로 포함되지 않는 29세 이하의 연령대와 결측치를 제외하고, 성향점수매칭을 통해 136,672명을 대상으로 선정하였다.

3. 변수 선정

1) 종속변수

종속변수는 암 검진 수진행동으로 최근 2년 동안의 암 검진(국가암검진 및 민간암검진)을 시행했을 경우에는 '예', 최근 2년 동안의 암 검진을 시행하지 않았을 경우는 '아니오'의 두 개 범주로 구분하였다.

2) 독립변수

독립변수는 인구사회학적 특성, 건강행태, 지역특성이다. 인구사회학적 특성에는 성별, 연령, 혼인상태, 학력, 가구소득, 고용형태 변수가 포함되었다[8, 11, 12, 14-16].

건강행태에는 음주, 흡연, 신체활동, 비만도, 만성질환 이환여부, 주관적 건강상태가 있다[8, 11, 15, 17].

지역특성은 시·군·구로 나누었다. 세종시는 여러 바이어스와 교란요인으로 작용할 가능성이 있어 본 연구의 분석에서 제외하였다[18, 19].

4. 분석 방법

본 연구의 목적에 따라 주요 관심 변수인 인구사회학적 특성 외에 존재할 수 있는 건강행태 요인과 지역 요인의 영향을 통제하기 위해 성향점수매칭을 적용하였다. 성향점수 매칭 전과 후에 각 요인별 암 검진 행위의 차이를 확인하기 위해 chi-square분석을 실시하였고, 암 검진 수진행동에 미치는 요인과 영향 정도를 파악하기 위해 로지스틱 회귀분석을 실시하였다.

1) 성향점수매칭

본 연구에서는 성향점수매칭 방법을 통하여 교란요인을 통제하였다. 일반적으로 두 집단을 비교하는 연구는 비무작위 표본 추출로 진행되며, 이때 선택편의 문제와 결과의 과대 혹은 과소 추정의 문제가 발생할 수 있다. 이에 연구 설계 단계에서 교란요인으로 작용할 수 있는 변수를 선정하고, 이를 공변량으로 산출한 후 실험군과 대조군 집단을 유사하게 만드는 성향점수매칭 방법이 소개되었다[20-22].

성향점수매칭 분석과정은 다음과 같다. 첫째, 실험군과 대조군의 성향점수를 추정한다. 본 연구의 종속변수는 암 검진 수진행동이며, 이에 영향을 미칠 것으로 예상되는 건강행태(음주, 흡연, 신체활동, 비만도, 만성질환 이환여부, 주관적 건강상태)와 지역 특성을 공변량으로 투입하여 성향점수를 추정하였다. 둘째, 추정된 성향점수 값을 비교하여 가장 유사한 성향점수를 가진 실험군과 대조군을 매칭한다. 본 연구에서는 그리디 매칭 방법(Greedy matching)을 사용하였다. 그리디 매칭 방법은 캘리퍼(Caliper)를 사용하여 실험군을 중심으로 일정한 성향점수의 범위를 설정하고, 이 범위에 해당하는 대조군의 개체들 중 가장 가까운 개체를 선택하는 방법이다[20]. 캘리퍼 허용오차는 정해진 기준은 없지만 통상적으로 0.01~0.00001이 사용되며, 캘리퍼 허용오차 크기가 0에 가까울수록 개체수가 적어지기 때문에 실험군의 표본 수를 고려하여 사용해야 한다[20]. 본 연구에서는 1:1 최근접 이웃방법(nearest neighbor matching)을 사용하였고, 캘리퍼는 0.0001 값을 주었으며, 매칭 결과 136,672명이 매칭 되었다. 셋째, 매칭 후 실험군과 대조군이 제대로 매칭 되었는지 확인한다. 본 연구에서는 실험군과 대조군의 공변량의 표준화된 차이와 그래프를 통해 매칭결과를 확인하였다. 매칭된 대상을 기준으로 chi-square 분석을 실시하였다[23].

2) 로지스틱 회귀분석

성향점수매칭 전후 연구대상자의 암 검진 수진행동에 미치는 인구사회학적 요인의 영향을 파악하기 위해 로지스틱 회귀분석(logistic regression analysis)을 실시하였다. 성향점수매칭은 SPSS ver. 23.0을 사용하였으며, 로지스틱 회귀분석은 SAS ver. 9.4을 이용하였다.

RESULTS

Volume: 40, Article ID: e2018011
https://doi.org/10.4178/epih.e2018011

1. 연구대상자의 일반적 특성에 대한 기술통계 분석

일반적으로 성향점수매칭이 잘 이루어졌는지 확인하기 위해 매칭 전후 각각 차이분석을 실시한다. <Table 1>은 연구대상자의 매칭 전후 차이분석에 대한 결과이다. 매칭 전 암 검진을 한 군은 115,665명, 암 검진을 하지 않은 군은 73,010명이었다. 건강행태(음주, 흡연, 신체활동, 비만도, 만성질환 이환여부, 주관적 건강상태)와 지역 특성을 공변량으로 투입하여 성향점수에 의해 1:1 매칭을 실시한 결과 암 검진을 받은 군과 암 검진을 받지 않은 군의 수는 각각 68,336명이 매칭 되었다. 매칭 전 유의한 차이를 보였던 음주, 흡연, 신체활동, 비만도, 만성질환 이환여부, 주관적 건강상태, 지역 변수는 매칭 후 모두 통계적으로 유의한 차이가 없는 것으로 나타나 매칭이 잘 이루어졌다는 것을 알 수 있다.

Table 1. General characteristics of study variables by cancer screening

	Variables	Cancer screening					
		Before PSM			After PSM		
		Yes (n=115,665)	No (n=73,010)	X^2	Yes (n=68,336)	No (n=68,336)	X^2
PS matching variables	**Alcohol drinking**						
	Current drinker	77,853(67.3)	50,813(69.6)	108.053***	46,820(68.5)	46,820(68.5)	0.000
	Non-drinker	37,812(32.7)	22,197(30.4)		21,516(31.5)	21,516(31.5)	
	**Smoking**						
	Current smoker	97,025(83.9)	52,270(71.6)	4,094.415***	51,946(76.0)	51,946(76.0)	0.000
	Non-smoker	18,640(16.1)	20,740(28.4)		16,390(24.0)	16,390(24.0)	
	**Physical activity**						
	Yes	100,595(87.0)	60,505(82.9)	602.557***	57,181(83.7)	57,181(83.7)	0.000
	No	15,070(13.0)	12,505(17.1)		11,155(16.3)	11,155(16.3)	
	**BMI group**						
	Underweight	4,655(4.0)	3,779(5.2)	206.896***	3,261(4.8)	3,404(5.0)	3.636
	Normal	50,812(43.9)	32,021(43.9)		30,089(44.0)	30,089(44.0)	
	Overweight	30,270(26.2)	17,727(24.3)		16,908(24.7)	16,908(24.7)	
	Obesity	29,928(25.9)	19,483(26.7)		18,078(26.5)	17,935(26.2)	
	**Chronic disease**						
	Yes	61,227(52.9)	46,956(64.3)	2,369.459***	42,537(62.2)	42,537(62.2)	0.000
	No	54,438(47.1)	26,054(35.7)		25,799(37.8)	25,799(37.8)	
	**Subjective health status**						
	Healthy	38,027(32.9)	26,767(36.7)	300.865***	24,472(35.8)	24,615(36.0)	1.124
	Average	51,674(44.7)	31,300(42.9)		29,329(42.9)	29,329(42.9)	
	Unhealthy	25,964(22.4)	14,943(20.5)		14,535(21.3)	14,392(21.1)	
	**Region**						
	City	33,524(29.0)	21,372(29.3)	102.252***	19,924(29.2)	19,924(29.2)	0.000
	County	39,911(34.5)	23,638(32.4)		22,409(32.8)	22,409(32.8)	
	District	42,230(36.5)	28,000(38.4)		26,003(38.1)	26,003(38.1)	
PS non-matching variables	**Gender**						
	Men	48,819(42.2)	39,166(53.6)	2,352.684***	31,576(46.2)	34,969(51.2)	337.168***
	Women	66,846(57.8)	33,844(46.4)		36,760(53.8)	33,367(48.8)	
	**Age (years)**						
	30~39	12,720(11.0)	20,789(28.5)	11063.150***	8,572(12.5)	18,904(27.7)	6,138.021***
	40~49	26,747(23.1)	16,162(22.1)		17,514(25.6)	14,832(21.7)	
	50~59	30,763(26.6)	13,297(18.2)		18,031(26.4)	12,512(18.3)	
	60~69	24,537(21.2)	9,006(12.3)		13,017(19.0)	8,732(12.8)	
	≥70	20,898(18.1)	13,756(18.8)		11,202(16.4)	13,356(19.5)	

Volume: 40, Article ID: e2018011
https://doi.org/10.4178/epih.e2018011

**Marital status**						
Married	94,512(81.7)	51,344(70.3)	6,955.404***	55,990(81.9)	48,321(70.7)	3,782.788***
Separated/Divorced/Bereaved	17,710(15.3)	12,527(17.2)		9,905(14.5)	11,922(17.4)	
Unmarried	3,443(3.0)	9,139(12.5)		2,441(3.6)	8,093(11.8)	
**Educational level**						
Completed primary school	30,281(26.2)	16,621(22.8)		16,138(23.6)	16,113(23.6)	
Completed middle school	16,724(14.5)	7,689(10.5)		9,344(13.7)	7,297(10.7)	
Completed high school	36,077(31.2)	23,811(32.6)	1,554.073***	22,155(32.4)	21,859(32.0)	530.219***
Completed college	9,525(8.2)	8,534(11.7)		6,060(8.9)	7,817(11.4)	
Completed university	18,936(16.4)	14,024(19.2)		12,105(17.7)	13,026(19.1)	
Completed graduated school	4,122(3.6)	2,331(3.2)		2,534(3.7)	2,224(3.3)	
**Monthly household income(10^3KRW)**						
<1,000	24,719(21.4)	16,129(22.1)		13,541(19.8)	15,426(22.6)	
1,000 ~ <2,000	20,314(17.6)	12,760(17.5)	621.613***	11,419(16.7)	11,940(17.5)	707.748***
2,000 ~ <3,000	20,756(17.9)	15,156(20.8)		12,515(18.3)	14,000(20.5)	
3,000 ~ <4,000	18,178(15.7)	12,199(16.7)		11,211(16.4)	11,315(16.6)	
4,000 ~ <5,000	12,643(10.9)	7,277(10.0)		7,855(11.5)	6,771(9.9)	
≥5,000	19,055(16.5)	9,489(13.0)		11,795(17.3)	8,885(13.0)	
**Employment type**						
Employer and self-employed	25,594(22.1)	15,613(21.4)	126.180***	15,469(22.6)	14,322(21.0)	102.067***
Salary	42,680(36.9)	28,815(39.5)		26,860(39.3)	26,327(38.5)	
Inoccupation	47,391(41.0)	28,582(39.1)		26,007(38.1)	27,687(40.5)	

Values are presented as number (%).
PSM, propensity score matching; PS, propensity score; KRW, Korean won.
***p<0.001.

<Figure 2>은 성향점수매칭이 잘 이루어졌는지 확인하기 위한 히스토그램으로 매칭 전(unmatched)과 매칭 후(matched)의 실험군(treated)과 대조군(control)의 성향점수 분포를 나타낸다. 매칭 후 암 검진을 받은 군과 암 검진을 받지 않은 군의 성향점수 분포가 비슷해졌음을 알 수 있다.

Volume: 40, Article ID: e2018011
https://doi.org/10.4178/epih.e2018011

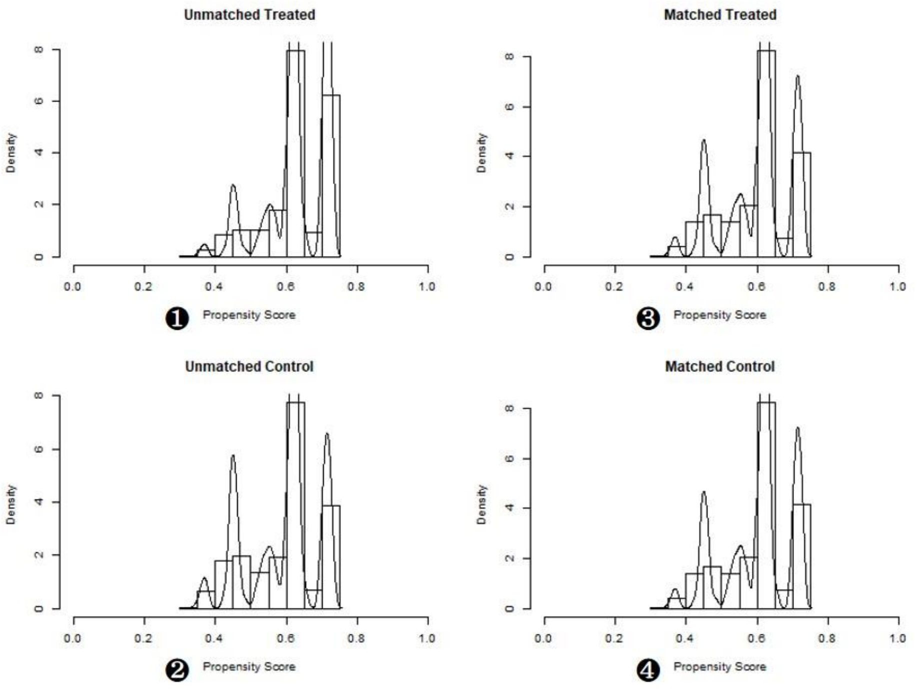

Figure 2. Comparison of propensity score distribution before and after propensity score matching. Treated: Cancer screening "Yes", Control: Cancer screening "No" (1) Unmatched treated. (2) Unmatched control. (3) Matched treated. (4) Matched control

2. 암 검진 수검여부를 종속변수로한 로지스틱 회귀분석

<Table 2>은 성향점수 매칭 전후 암 검진 수진 여부를 종속변수로 하여 인구사회학적 특성, 건강행태 그리고, 지역 변수를 독립변수로 포함한 로지스틱 회귀분석의 결과이다(Wald test for Global Null Hypothesis X^2=6722.866, $p<0.0001$(before PSM), X^2=9516.227, $p<0.0001$(after PSM)). AIC 및 C 통계량을 근거로 최종 모델을 선정하였으나, Hosmer-Lemeshow test에서는 모델이 적합하지 않은 것으로 나타났다($p<0.0001$). 이는 극단적으로 큰 표본에서는 예측치와 관측치 간의 차이가 작기 때문에 Hosmer-Lemeshow test가 유의하게 나타난 것으로 볼 수 있다 [24].

성향점수 매칭 전 분석 결과에서는 남자가 암 검진을 할 확률이 39.0% 낮게 나타났으며, 70세 이상에 비해 50대, 60대는 암 검진을 확률이 더 높았고, 30대, 40대에서는 암 검진을 확률이 낮게 나타났다. 혼인상태의 경우에는 미혼자에 비해 혼인한 경우는 2.56배, 별거, 이혼, 사별의 경우는 약 1.62배 검진할 가능성이 높다는 결과를 보였다. 또한 교육수준, 월 소득이 낮을수록 암 검진을 할 확률이 낮게 나타났으며, 무직(주부, 학생 포함)의 경우보다 고용주 및 자영업자에서 1.16배, 임금노동자 1.35배로 암 검진을 받을 확률이 높게 나타났다.

또한, 암 검진의 수진 확률은 현재 음주를 하는 경우 1.15배, 운동을 하는 경우 1.13배 높게 나타났으며, 현재 흡연을 할 경우 35% 낮게 나타났다. BMI 정상군보다 저체중, 비만군이 암 검진할 확률이 각각 9.0%, 4.0% 낮았고, 만성 질환이 있는 군에서 암 검진을 받을 확률이 1.26배 높게 나타났다. 건강상태를 보통으로 평가한 경우가 좋다고 평가한 경우보다 암 검진을 받을 확률이 더 높았다. 지역특성의 경우 구지역에 거주하는 사람에 비해 시와 군에 거주하는 사람이 암 검진을 받을 확률이 더 높은 것으로 나타났다.

성향점수 매칭 후 분석 결과에서는 여자보다 남자가 암 검진을 할 확률이 38.0% 낮게 나타났으며, 70세 이상에 비해 50대, 60대는 암 검진을 확률이 더 높았고, 30대, 40대에서는 암 검진을 확률이 낮게 나타났다. 혼인상태의 경우에는 미혼자에 비해 혼인한 경우는 2.50배, 별거, 이혼, 사별의 경우는 약 1.62배 검진할 가능성이 높다는 결과를 보였다. 또한 교육수준, 월 소득이 낮을수록 암 검진을 할 확률이 낮게 나타났으며, 무직(주부, 학생 포함)의 경우보다 고용주 및 자영업자에서 1.15배, 임금노동자 1.36배로 암 검진을 받을 확률이 높게 나타났다.

암 검진의 수진 확률은 현재 음주를 하는 경우와 현재 흡연을 할 경우 각각 1.07배, 1.30배 높게 나타났으며, 운동을 할 경우에는 6% 낮게 나타났다. BMI 정상군보다 저체중, 비만군이 암 검진할 확률이 약간 높았으며, 만성 질환이 있는 군에서 암 검진을 받을 확률이 20.0% 낮게 나타났다. 건강상태를 나쁘다고 평가했을 때 암 검진을 받을 확률은 1.11배로 보통으로 평가한 경우 보다 약간 높았다. 지역특성의 경우 통계적으로 유의한 차이가 없었다.

Table 2. Results of the logistic regression analysis

Variables	OR (95% CI)	
	Before PSM[1]	After PSM[2]
**Gender** (ref.=women)		
Men	0.61(0.59-0.63)***	0.62(0.6-0.63)***
**Age (years)** (ref.=≥70)		
30~39	0.31(0.29-0.32)***	0.30(0.28-0.32)***
40~49	0.78(0.75-0.82)***	0.76(0.72-0.80)***
50~59	1.12(1.08-1.17)***	1.07(1.03-1.12)**
60~69	1.50(1.45-1.55)***	1.45(1.39-1.51)***
**Marital status** (ref.=unmarried)		
Married	2.56(2.45-2.68)***	2.50(2.38-2.63)***
Separated/divorced/bereaved	1.62(1.54-1.71)***	1.62(1.53-1.72)***
**Educational level** (ref.=completed graduated school)		
Completed primary school	0.65(0.61-0.69)***	0.66(0.62-0.71)***
Completed middle school	0.77(0.73-0.83)***	0.78(0.73-0.84)***
Completed high school	0.74(0.70-0.79)***	0.75(0.70-0.80)***
Completed college	0.82(0.77-0.87)***	0.81(0.76-0.87)***
Completed university	0.87(0.82-0.93)***	0.88(0.83-0.94)***
**Monthly household income (10^3 Korean won)** (ref.=≥5,000)		
<1,000	0.70(0.67-0.73)***	0.69(0.66-0.72)***
1,000 ~ <2,000	0.73(0.70-0.76)***	0.71(0.68-0.74)***
2,000 ~ <3,000	0.75(0.72-0.77)***	0.74(0.71-0.77)***
3,000 ~ <4,000	0.82(0.79-0.85)***	0.81(0.78-0.85)***
4,000 ~ <5,000	0.93(0.89-0.96)***	0.92(0.88-0.96)***
**Employment type** (ref.= inoccupation)		
Employer and self-employed	1.16(1.13-1.20)***	1.15(1.12-1.19)***
Salary	1.35(1.31-1.39)***	1.36(1.32-1.40)***
**Alcohol drinking** (ref.=non-drinker)		
Current drinker	1.15(1.13-1.18)***	1.07(1.05-1.10)***
**Smoking** (ref.=non-smoker)		
Current smoker	0.65(0.64-0.67)***	1.30(1.26-1.34)***
**Physical activity** (ref.=no)		
Yes	1.33(1.29-1.37)***	0.94(0.91-0.97)***

Volume: 40, Article ID: e2018011
https://doi.org/10.4178/epih.e2018011

**BMI group** (ref.=normal)		
Underweight	0.91(0.87-0.95)***	1.10(1.05-1.16)***
Overweight	1.00(0.97-1.02)	0.96(0.93-0.99)**
Obesity	0.96(0.93-0.98)***	1.05(1.02-1.08)***
**Chronic disease** (ref.=n o)		
Yes	1.26(1.23-1.30)***	0.80(0.77-0.82)***
**Subjective health status** (ref.=average)		
Healthy	0.94(0.92-0.96)***	1.02(0.99-1.05)
Unhealthy	1.01(0.98-1.04)	1.11(1.07-1.14)***
**Region** (ref.=district)		
City	1.08(1.06-1.11)***	1.01(0.99-1.04)
County	1.13(1.10-1.16)***	1.00(0.98-1.03)

Values are presented as odds ratio (95% confidence interval).
PSM, propensity score matching; KRW, Korean won.
**p<0.01, ***p<0.001.
[1]c-statistics=0.683, Hosmer-Lemeshow goodness-of-fit test p<0.001; [2]c-statistics=0.653, Hosmer-Lemeshow goodness-of-fit test p<0.001.

DISCUSSION

1. 연구자료 및 방법에 대한 고찰

본 연구는 2014년 자료를 이용하였다. 암 검진에 대한 선행연구는 대부분 특정 암[11,13, 14] 또는 일부 지역 주민을 대상으로 연구되어[8, 10, 14, 15, 25], 우리나라 국민의 전반적인 암 검진 수진요인을 파악하는데 어려움이 있다. 따라서 본 연구는 지역 간 비교가 가능하도록 조사체계를 표준화한 지역사회건강조사 자료를 이용하여 전국을 대상으로 전반적인 암 검진 수진 현황을 살펴보았다는 데에 의의가 있다.

PSM은 성향점수를 통해 연구대상이 되는 그룹들의 대상자들 사이에 관찰된 공변량들의 균형을 맞추어줌으로써 무작위배정 연구와 같은 상황을 만들어주는 방법이다[26]. 이는 암 검진 수진행동의 영향요인에 대한 기존 연구에서 많이 사용하지 않았던 방법으로 본 연구는 PSM을 활용하여 선택편향과 교란요인을 최소화하여 암 검진 수진행동의 영향요인을 더 명확하게 확인할 수 있다는 장점이 있다.

2. 연구결과에 대한 고찰

성향점수 매칭을 전후 로지스틱 회귀분석을 실시하였다. 성향점수 매칭 변수 중 흡연, 만성질환 보유, 지역 변수는 PSM 전후 결과의 차이가 있었다.

인구사회학적 특성에서는 PSM 전후의 로지스틱 회귀분석 결과가 유사하게 나타났다. 성별의 경우 기존연구[5, 8, 25, 27-29]와 마찬가지로 여성이 남성보다 암 검진 수검률이 높았으며, 남자의 수검확률이 약간 더 높은 것으로 나타난 위암 검진 영향요인에 대한 연구인[30, 31] 의 결과와는 일치하지 않았다. 이는 남성의 더 많은 사회참여로 인한 시간부족, 암 검진에 대한 인지도와 실천의지가 낮은 것으로 설명할 수 있으며, 국가 암 검진 항목 중 여성에게만 해당되는 유방암과 자궁경부암이 포함되어 있어 상대적으로 여성이 더 많은 검진 기회를 갖기 때문으로 볼 수 있다

[25, 27] 직장 검진의 적극적 활용과 전립샘 암과 같은 남성 대상 암 검진 항목의 추가 도입 등을 통해 남성의 암 검진 수검률 향상을 꾀할 수 있을 것으로 사료된다.

연령에서는 연령이 증가할수록 암 검진 확률이 더 높게 나타나 기존 연구[5, 11, 25, 27, 29, 32]와 일치하였다. 이는 연령이 증가 할수록 만성질환 노출이 많아 의료 이용의 기회가 많고 그를 통해 건강관리와 검진에 대한 정보 습득으로 암 검진에 대한 인지도와 실천도가 높아진 것으로 설명할 수 있다[27]. 또한 70세 이상에서 상대적으로 암 검진의 교차비가 낮은 것은 70세 이상에서는 암 검진에 대한 수요와 편익이 상대적으로 적어 암 검진에 참여할 가능성이 낮기 때문으로 보인다[25].

혼인상태의 경우 대부분의 연구[8, 11, 15, 25, 27, 30, 32-34]에서 배우자 있을 때 배우자가 없는 경우 보다 암 검진 수검률이 높게 나타났으며, 본 연구 결과도 이와 일치하는 경향을 보였다. 미혼에 비해 혼인뿐만 아니라 사별, 이혼 등의 경우에도 암 검진율이 높은 것으로 나타났는데 이를 통해 배우자 또는 가족의 지지와 관심이 암 검진 수검에 영향을 미치는 요소라고 판단할 수 있다. 교육수준과 월 가구 소득이 낮을수록 암 검진 확률이 낮은 것으로 나타났는데 이는 기존의 연구결과[8, 11, 15, 29, 30, 32, 33]와 일치하였다. 따라서 암 검진에 있어서도 형평성 제고를 위한 정책적 노력이 필요함을 시사한다.

대부분의 연구에서는 고용형태를 포함하지 않거나 통계적 차이를 보이지 않았는데[5, 8, 10, 11, 25, 29, 34], 본 연구에서는 무직 보다 직업이 있는 경우(임금노동자, 자영업자, 가족무급종사자)가 암 검진 수검 확률이 높게 나타났으며, 이는 Kim YB et al.(2000)[35]의 자궁경부암, 유방암 연구와 차이를 보였다. 이는 연구 대상자를 여성으로 한정했을 경우에는 시간적 여유가 암 검진에 상대적으로 더 많은 영향을 미치는 것으로 볼 수 있으며, 남녀 전체를 대상으로 했을 때는 직장 검진의 제공 등을 통한 의료기관 접근성 향상과 암 검진 정보 습득 기회의 증가 등이 암 건진 수검 확률에 영향을 미친 것으로 생각된다.

다음으로 건강행태를 살펴보면, 현재 음주를 할 경우는 PSM 전후 각각 1.15배, 1.07배로 암 검진 확률이 높게 나타났으며, 현재 흡연의 경우 PSM 전에는 35.0% 낮게, 후에는 암 검진을 받을 확률이 1.30배 높게 나타났다. 기존 연구에서 음주, 흡연과 같은 건강행태가 암 검진 수진행동에 미치는 영향은 다양한 결과를 보였다[5, 28, 30, 33, 34]. 음주와 흡연은 잘 알려진 암의 위험요인이므로 바람직하지 못한 건강행태를 스스로 인지하고 있기 때문에 현재 음주와 현재 흡연 군에서 암 검진 확률이 높게 나타난 것으로 해석할 수 있으며, 이는 성향점수 매칭을 통해 보정이 잘 이루어진 것으로 볼 수 있다. 비만도는 매칭 전에는 정상 체중에 비해 저체중과 비만에서 암 검진을 할 확률이 낮게 나타났으며, 매칭 후에는 암 검진을 할 확률이 높게 나타났다. 이는 Park JD et al.(2004)[34]의 연구, Fagan et al.(2011)[36]의 남성 대장직장암, 전립샘 검진 결과와 일치하였으며, Fagan et al.(2011)[36] 연구의 자궁경부암 검진 결과와는 불일치하였다. 저체중과 비만의 경우 본인이 건강하지 않다고 느끼기 때문에 암 검진에 더 많이 참여한다고 추정할 수 있다.

만성질환 이환 여부와 주관적 건강상태도 PSM 전후 차이를 보였다. PSM 후의 결과가 만성 질환이 있을 경우 일반적으로 정기적인 병원 방문으로 의료기관 이용도가 높아 암 검진을 많이 받는 다는 기존의 연구[8, 34, 36]와 일치하여, 보정이 잘 되었음을 보여준다. 주관적 건강상태는 암 검진 수검요인 연구[8], 암 생존자 대상 연구[27, 33], 경상남도 지역의 암 검진 연구인 Kim RB et al.(2010)[28]의 연구에서는 유의한 차이를 보이지 않았는데 본 연구에

Volume: 40, Article ID: e2018011
https://doi.org/10.4178/epih.e2018011

서는 PSM 후에 건강상태가 나쁘다고 평가한 경우에 암 검진을 받을 확률이 1.11배 높은 것으로 나타났다. 이는 본인의 건강에 대한 염려 때문에 암 검진에 더 많이 참여하는 것으로 생각할 수 있다.

지역특성을 살펴보면, PSM 전에는 구지역에 거주하는 사람에 비해 시와 군지역에 거주하는 사람이 암 검진을 받을 확률이 높게 나타났으나 PSM 후에는 유의한 차이를 보이지 않았다. 지역 간 격차가 나타나지 않은 것은 다양한 의료기관에서 암 검진이 가능해져 접근성 문제가 일부 해소되었기 때문으로 설명할 수 있다[25].

연구결과의 정책적 시사점은 다음과 같다. 첫째, 암 검진의 수검률을 높이기 위하여 암 검진에 대한 의도를 높여야 한다. 암 검진 의도는 암 검진 수진행동의 강력한 영향요인으로 볼 수 있다[8]. 개인의 암 검진 의도를 높이기 위해서는 교육을 통해 검진을 통한 암 조기발견이 암 예방과 생존율 향상에 중요하다는 것을 알려야 한다. 또한 다양한 홍보채널과 보건교육을 통해 암 검진에 대한 인식을 개선하는 것이 필요하다. 둘째, 암 검진 수검률은 인구사회학적 요인인 교육수준, 월 가구소득, 고용형태에 따라 차이를 보이는데 이를 해결하기 위해 다빈도 암종의 무상검진 확대, 유급휴가 제공 등과 같은 암 검진을 쉽게 받을 수 있는 환경 조성과 함께 소득과 교육 수준이 낮은 인구집단을 대상으로 한 암 검진 홍보와 교육이 필요하다. 셋째, 암은 정기적으로 검진이 필요한 질병으로 재수검을 높이는 것이 중요하다. Kim HJ(2015)[37]의 연구에 의하면, 암 검진에 대한 만족도가 높을수록 암 검진 재수검 의도가 높았다. 재수검 대상자의 수검률을 높이기 위해 암 검진의 질을 향상시킬 필요가 있으며, 일률적으로 제공하는 암 검진 프로그램에서 벗어나 개인의 인구사회학적 특성과 행태에 기초한 개인 맞춤형 암 검진 프로그램을 개발하는 등 다양하고 차별화된 정책 및 제도의 보완이 필요할 것이다.

본 연구의 제한점으로는 첫째, 지역사회건강조사 자료의 암 검진 여부 변수에는 다른 국가암검진과 민간암검진 모두를 포함하고 있어 연구 대상자들이 받은 암 검진의 내용과 특성이 다를 수 있다. 둘째, 본 연구는 단면조사 연구인 지역사회건강조사 자료를 이용한 연구로서 변수들 간의 관련성은 파악할 수 있었지만 인과관계를 파악하는 데는 어려움이 있다. 추후 코호트 자료나 패널 자료 등을 활용해 변수들 간의 인과성을 파악해 볼 필요가 있다. 셋째, 2차 조사 자료를 이용하였기 때문에 원 자료에서 제공하지 않는 암 검진에 영향요인으로 알려진 암 가족력[30, 38], 개인력[29, 30] 등의 변수는 파악할 수 없었다. 추후 연구에서는 기존에 암 검진 영양 요인으로 알려진 모든 변수를 포함해 분석함으로써 더 명확한 암 검진 수진 영향 요인들을 파악할 수 있을 것이다. 넷째, 지역사회건강조사는 가구 방문 1:1 면접조사로 자료를 수집함에 따라 암 검진과 인구사회학적 변수 관련 문항 조사에 있어 일부 편견이 개입되었을 수 있으며, 조사가 대상자의 기억에 의존하여 이루어지므로 회상오류(recall bias) 발생 가능성이 있다. 따라서 암 검진 관련 의료이용, 병원기록 등을 연계하여 분석하는 방법으로 이를 보완할 수 있을 것이다.

본 연구는 2014년 지역사회건강조사 자료를 바탕으로 만 30세 이상의 성인의 암 검진 수진행동에 영향을 미치는 인구사회학적, 건강행태, 지역 요인을 분석하였다. 연구결과 성별, 연령, 혼인상태, 교육수준, 월 가구소득, 음주, 흡연, 비만도, 만성질환 이환여부, 주관적 건강상태 변수가 암 검진 수진행동의 영향요인으로 나타났다.

암은 조기에 발견할수록 생존율이 높기 때문에 암의 조기발견을 위한 암 검진은 중요하다. 따라서 암 검진 수진행동을 높이기 위해서는 교육을 통해 암 검진에 대한 중요성을 인식시킬 필요가 있으며, 다양한 홍보채널을 활용해 암 검진 참여율을 높여야 한다. 또한 암은 주기적인 검진이 필요한 질병이므로 재수검을 높이기 위해 일률적으로 제공하는 암 검진 프로그램 외에 개인의 인구사회학적, 건강행태와 연계한 개인 맞춤형 암 검진 프로그램을 개발하고 확대하는 것이 바람직할 것으로 사료된다.

Volume: 40, Article ID: e2018011
https://doi.org/10.4178/epih.e2018011

본 연구는 지역 간 비교가 가능하도록 설계된 자료를 이용하여 우리나라 전체 인구를 대상으로 암 검진 수진행동에 미치는 영향을 파악하였으며, PSM을 통해 교란요인을 통제하여 암 검진 수진행동의 영향요인을 보다 명확하게 분석하였다는 점에서 의의가 있다.

Volume: 40, Article ID: e2018011
https://doi.org/10.4178/epih.e2018011

REFERENCES

1. World Health Organization. Cancer; 2017 [cited 2017 Dec 28]. Available from: http://www.who.int/mediacentre/factsheets/fs297/en/.

2. Jung KW, Won YJ, Oh CM, Kong HJ, Lee DH, Lee KH, et al. Cancer statistics in Korea: incidence, mortality, survival, and prevalence in 2014. Cancer Res Treat 2017;49:292-305.

3. Statistics Korea. Cause of death 2017 [cited 2018 Jan 3]. Available from: http://www.index.go.kr/potal/stts/idxMain/selectPoSttsIdxSearch.do?idx_cd=1012 (Korean).

4. Statistics Korea. Cause of death statistics in 2016, 2017 [cited 2017 Dec 28]. Available from: http://www.kostat.go.kr/portal/korea/kor_nw/2/1/index.board?bmode=read&aSeq=363268 (Korean).

5. Roh WN, Lee WC, Kim YB, Park YM, Lee HJ, Meng KH. An analysis on the factors associated with cancer screening in a city. Korean J Epidemiol 1999;21:81-92 (Korean).

6. Jung KW, Won YJ, Kong HJ, Oh CM, Shin A, Lee JS. Survival of Korean adult cancer patients by stage at diagnosis, 2006-2010: national cancer registry study. Cancer Res Treat 2013;45:162-171.

7. World Health Organization. Cancer prevention [cited 2018 Jan 21]. Available from: http://www.who.int/cancer/prevention/en/.

8. Kwak MS, Park EC, Bang JY, Sung NY, Lee JY, Choi KS. Factors associated with cancer screening participation, Korea. J Prev Med Public Health 2005;38:473-481 (Korean).

9. Suh M, Song S, Cho HN, Park B, Jun JK, Choi E, et al. Trends in participation rates for the national cancer screening program in Korea, 2002-2012. Cancer Res Treat 2017;49:798-806.

10. Kim YB, Lee WC. A study on the behavioral factors related to stomach cancer screening among adults in a rural area. Korean J Epidemiol 1999;21:20-30 (Korean).

11. Lee MH, Kim SH. Breast cancer screening rates-related factors Korea women ever considering area environmental characteristics: the fourth Korea National Health and Nutrition Examination Survey (KNHANES IV). J Digit Converg 2014;12:437-449 (Korean).

12. Cho IS, Park YS. A study on regular cervical cancer screening behavior among middle-aged women. J Korean Acad Nurs 2004; 34:141-149 (Korean).

13. Ham OK. Analysis of factors related to mammography screening behavior women: use of health belief model. J Korean Public Health Nurs 2004;18:187-194 (Korean).

14. Kim YI, Cho YC. The associated factors of health examinations behaviors among some elderly persons in urban and rural areas. J Agric Med Community Health 2004;29:1-14 (Korean).

15. Kim MK, Nam EW, Jin KN. The factors influencing the odds of cancer examination in Wonju city. Korean J Health Educ Promot 2006;23:85-97 (Korean).

16. Lee JW, Ahn SY, Kim KH. Study on factors that influence cancer screening rate in urban and rural areas. J Digit Converg 2012;10: 269-278 (Korean).

17. Jeong GB, Park JY, Kwon SY, Park KO, Park PS, Park MY. A comparison between asia-pacific region criteria and entropy model criteria about body mass index of elderly females using morbidity of chronic disease. Korean J Community Nutr 2014;19:490-498 (Korean).

18. Shin SD. It came to the health checkup season: where are the citizens of Sejong? Goodmorning Chungcheong; 2015 Nov 25 [cited 2017 Dec 9]. Available from http://www.goodmorningcc.com/news/articleView.html?idxno=34693 (Korean).

19. Yoon S. Sejong city health center conducts cancer screening and health check-up. Aju Business Daily; 2015 Feb 8 [cited 2017 Feb 9]. Available from http://www.ajunews.com/view/201502082115 30589 (Korean).

20. Kim SY, Baek JI. FUZZY matching using propensity score: IBM SPSS 22 Ver. J Korean Data Inf Sci Soc 2016;27:91-100 (Korean).

21. Austin PC. Propensity-score matching in the cardiovascular surgery literature from 2004 to 2006: a systematic review and suggestions for improvement. J Thorac Cardiovasc Surg 2007;134:1128-1135.

22. Choi SJ, Kim SS. The analysis for effect on government R&D subsidies by using PSM. J Korea Acad Ind Coop Soc 2009;10:200-208 (Korean).

23. Seo EW, Lee KS. Difference in healthcare utilization for percutaneous transluminal coronary angioplasty inpatients by insurance types: propensity score matching analysis. Health Policy Manag 2015;25:3-10 (Korean).

24. Feudtner C, Hexem KR, Shabbout M, Feinstein JA, Sochalski J, Silber JH. Prediction of pediatric death in the year after hospitalization: a population-level retrospective cohort study. J Palliat Med 2009;12:160-169.

25. Sung NY, Park EC, Shin HR, Choi KS. Participation rate and related socio-demographic factors in the national cancer screening program. J Prev Med Public Health 2005;38:93-100 (Korean).

26. Jang EJ, Ahn J, Jung SY, Hwang JS, Lee JY, Shim JI. Methods for the control of measured confounders in outcomes research. Seoul: National Evidence-based Healthcare Collaborating Agency; 2013, p. 1-272 (Korean).

27. Yang SE, Han NK, Lee SM, Kim TH, Chung W. The factors related to the non-practice of cancer screening in cancer survivors: based on the 2007-2012 Korean National Health and Nutrition Examination Survey. Health Policy Manag 2015;25:162-173 (Korean).

28. Kim RB, Park KS, Hong DY, Lee CH, Kim JR. Factors associated with cancer screening intention in eligible persons for national cancer screening program. J Prev Med Public Health 2010;43:62-72 (Korean).

29. Cullati S, Charvet-Bérard AI, Perneger TV. Cancer screening in a middle-aged general population: factors associated with practices and attitudes. BMC Public Health 2009;9:118.

30. Kim HK. Influence of socioeconomic status and health behavior with cancer screening program [dissertation]. Seoul: Yonsei University; 2006 (Korean).

31. Shin JY, Lee DH. Factors associated with the use of gastric cancer screening services in Korea: the Fourth Korea National Health and Nutrition Examination Survey 2008 (KNHANES IV). Asian Pac J Cancer Prev 2012;13:3773-3779.

32. Kwon YM, Lim HT, Lee K, Cho BL, Park MS, Son KY, et al. Factors associated with use of gastric cancer screening services in Korea. World J Gastroenterol 2009;15:3653-3659.

33. Cho J, Guallar E, Hsu YJ, Shin DW, Lee WC. A comparison of cancer screening practices in cancer survivors and in the general population: the Korean National Health And Nutrition Examination Survey (KNHANES) 2001-2007. Cancer Causes Control 2010;21:2203-2212.

34. Park JD, Kang PS, Lee KS. Factors related with screening behaviors of breast and cervical cancers among women in a rural area. J Korean Soc Matern Child Health 2001;8:49-64 (Korean).

35. Kim YB, Ro WO, Lee WC, Park YM, Meng KH. The influence factors on cervical and breast cancers screening behavior of women in a city. J Korean Soc Health Educ Promot 2000;17:1-16 (Korean).

36. Fagan HB, Wender R, Myers RE, Petrelli N. Obesity and cancer screening according to race and gender. J Obes 2011;2011:218250.

37. Kim HJ. Factors influencing cancer rescreening intention of Korean elderly. Korean J Rehabil Nurs 2015;18:118-127 (Korean).

38. Bostean G, Crespi CM, McCarthy WJ. Associations among family history of cancer, cancer screening and lifestyle behaviors: a population-based study. Cancer Causes Control 2013;24:1491-1503.
